# Dr. Symonds on the Powers of Nature in the Cure of Diseases

**Published:** 1847-04

**Authors:** J. A. Symonds


### III. On the Powers of Nature in the Cure of Diseases. In Reply to Dr. Combe.

By J. A. Symonds, M.D. etc.

(*In a Letter to John Forbes, M.D., F.R.S.*)

Bristol, March 1, 1847.

My dear Sir,—I am glad that Dr. Combe has honoured my letter with his comments, because they give me an opportunity of correcting a misapprehension into which he has fallen, and at the same time of enforcing some opinions which I have already expressed. In the remarks I am about to make I hope to emulate the good spirit in which I believe Dr. Combe's letter to have been written, although I shall not hesitate to allow myself an almost equal latitude of criticism.

Dr. Combe is in error when he supposes that my letter to you was intended to be a reply to his communication. Had he read it carefully and with a distinct remembrance of your Essay, he would have seen that my remarks were directed against such injurious inferences as seemed likely to be drawn from your paper. For after expressing my satisfaction that you had yourself made a statement corrective of the misunderstanding occasioned by your first paper, I alluded respectfully and in general terms to Dr. Combe's and certain anonymous epistles, which appeared to me "to set rather too much in the direction of the expectant method." All that I said bearing specifically upon Dr. Combe was at the close of my letter, and had reference to his suggestion of an experimental investigation of the claims of homœopathy. And as I shall have no better opportunity in the present letter, I must in this place observe that he has rather evaded than answered my question: "What is to induce us to administer a scruple of jalap for a looseness, and a grain or two of opium for a lethargy?" If there be truth in the similia-similibus doctrine, opium which causes a lethargy ought to cure one. To me it seems trifling with the subject to say, "See whether the medicine which cures a disease will produce it in a sound person." This was not the method of the homœopathists. If so, what an immense amount of chance experiments must have been made! to say nothing of their superfluousness. For if the drugs had really cured the various diseases in the homœopathic nosology, it would be useless to try their morbific qualities in order to establish their healing virtues. The line really pursued was that intimated in my question, which question I was justified in putting to any one who recommended us to test the like-by-like doctrine.

Dr. Combe endeavours to elude the difficulty by saying that he proposed a method just the reverse; forgetting that the fallacies would be much greater. That a particular medicine cures a particular disease may be true of quinine and ague, and of one or two others, but our trials would be few indeed if reduced to specifics. We do, however, know the physiological action of medicines. If opium narcotizes it ought to cure a coma. Dr. Combe himself says, "having tried their action in health, try the same remedies *in the usual doses* in the treatment of disease, with as much care and discrimination as possible, and record the results." Now the action of opium has been tried over and over again in health, and its effects are so similar to those of disease, that a case every now and then occurs in which it is very difficult to distinguish between them. Are you then prepared to give this drug, with never so much care and discrimination, in your next case of simple apoplexy? But I will not press this point further; I am sure Dr. Combe is too good a practitioner to let his catholicity towards the homœopathists induce him to try dangerous experiments by the bed-side.

To return to his critique. He says, "it seems to me a wise precaution, before proceeding to refute a supposed antagonist by either argument or ridicule, to ascertain very clearly whether a difference exists, and if so, in what it really consists." (p. 257). Most wise indeed! but, alas! for human consistency. The nearer the church the farther from God! For in the very next sentence he declares that I "condemned as wholly inert and absurd plans of treatment conducted in accordance with Nature." I have sought in vain throughout my brief letter for anything approaching to a warrant or excuse or even colour for this sweeping charge. So that I must infer that Dr. Combe so fully relied on the general truth of his maxim, and was so conscious of his habitual attention to it, that unfortunately for me in this particular instance he did not think it worth while to put it in practice; or perhaps he thought "exceptio probat regulam." To condemn treatment in accordance with Nature, would be to condemn what one has been learning and teaching all the days of one's medical life. For in what does it consist? *Generally*, in being founded on what is known of the structure and functions of the human body, aud *particularly* in the encouragement of the natural tendencies to reparation. Who has not been taught this rudimentary information? Surely we need no ghost to arise and tell us this. I am happy to say that it does not fall to my lot to meet with practitioners so grossly ignorant, that if a bronchitis were relieving itself by free expectoration they would try to check the secretion,—or if intestinal hemorrhage ensued on congestion of the liver, would administer astringents; while, on the other hand, were Nature allowing a delicate elderly patient to be worn down with bronchorrhea dependent on a relaxed state of the mucous membrane, they would not hesitate to give him sulphate of zinc, and if the intestinal bleeding proceeded from a follicular ulcer they would take care that sugar of lead or oil of turpentine should be applied to the bleeding surface as fast as possible, many an unhappy patient having died of this natural method of relief.

Dr. Combe avers that I seldom make "any special reference to the standard of Nature." I beg his pardon; I repeatedly referred to Nature, but not merely to *reparative* Nature, which seems to be the only nature in Dr. Combe's kingdom; I took some pains to direct attention to *morbific* Nature. He intimates that I am not "familiar with the study of Nature as an *aim*;" by which I presume he means as an *end*. Certainly I have studied it chiefly as a *mean* to the cure, and relief, and prevention of diseases. But it is hard to conjecture what exactly Dr. Combe intends by advocating so emphatically the observation of Nature in the study and treatment of disease. Surely, it is rather late in the day for a clear-headed, well-informed writer like Dr. Combe to preach "Homo naturæ minister et interpres." Is it not as if some professor should go to Oxford, and tell the men who are construing Juvenal and Æschylus, that he hopes they have learned their accidence? Study Nature! Talk prose! Have we not been doing it all our lives? What were those curious inquiries into the roots and juices of plants in our early days—those porings over muscles and nerves, vessels and viscera, in vaulted cellars,—those hospital walkings and dispensary trudgings,—those clinical labours, when, from the initial rigor to the terminal rhonchus, every phase of the case was carefully observed and faithfully depicted in those long, ledger-like note-books, and then the work of Nature further studied in the mortuary, with all aids and appliances from scalpel, scales, and measure;—and, since then, the night-watchings many by the bedside, and the reflections of sleepless hours on the day's observations,—and the endless interrogations of Nature, if haply one might attain to such knowledge of her doings, that here one might aid them, and there control or repress them? Or, to put individual experience aside, what have been the labours of the Baillies, the Laennecs, the Andrals, the Louis, the Brights, the Carswells, the Hasses, and Rokitanskis, and Vogels, and a host of recent histologists?—painful searchers all into the dark mysteries of Nature! Physiology is good, but it is not the one thing needful. There is pathology also—there is a dark as well as a bright side of Nature. It has pleased Infinite Power and Goodness to mix up evil with good in this world, and to place man in it with instinctive and other instructions, to do all he can towards extirpating or abating the one, and fostering and increasing the other, and to hope for a better state of being in which the sad enigma will be unriddled, and partial ill be resolved into universal good. Study Nature! yes—but let it be no narrow, one-sided study! Measure the *vis vitiatrix* as well as the *vis medicatrix!* It is better to be on our legs facing evil, defying it, and wrestling with it, than to sit simpering in the easy chair of optimism and crying "Peace, peace—when there is no peace."

Misled by his fancy that my letter was a critique upon his own, Dr. Combe repeatedly alleges, that I regard a method of treatment in accordance with Nature as equivalent to doing nothing at all. But *you* no doubt perceived that I was really inquiring whether diseases might be left to take their own way, a question naturally suggested by your original article, in which you had made a vigorous and able reclamation for the somewhat neglected *vis medicatrix*. After exposing the absurdity and nullity of the homœopathic system, you asked, how are the apparent cures under this system to be accounted for? And you concluded very reasonably, that, if the cases had got well, they had got well of themselves—not from the interposition of what Dr. Combe chooses to call "his natural treatment," but from no treatment at all. In my remarks I said, that if the statements of homœopathic success could be depended upon, they were most momentous; and the question which Dr. Combe thinks so superfluous, was inevitable. But I soon satisfied myself from an analysis of Dr. Fleischmann's table, that there was no need for any concern about this document and the possible inferences from it, and my conclusion was subsequently confirmed by Dr. Balfour's excellent Report. I then discussed the question on other grounds, and endeavoured to show, not only that Nature is often inadequate to the cure of diseases, but also that she is often doing mischief. Among the instances I adduced was that of sympathetic disorders in dentition, upon which Dr. Combe thus comments: "In teething, *Nature's efforts* are directed to effecting a passage outwards for the advancing tooth. If, from any circumstance, she proves unable for the effort, sound principle would assuredly direct us to *aid her* in it, and remove the obstacles which obstruct her progress; and I am at a loss to know what more rational or direct *aid* we could lend her in her own way, than making the opening through the gum, which she has failed to accomplish for herself." (p 259.)

It is remarkable that men of "strong sense," but "blinded by a preconceived notion," may fancy that they are dealing with facts when they are only using metaphors; for such are these phrases, "Nature's efforts," "failing to accomplish for herself," &c.—convenient enough as phrases, if we are not misled by them. Now, in this case, the practitioner who recognizes the cause of the illness and removes it, may if you please be acting "in accordance with Nature," but the plain fact is, that he knows his work and does it. As the tooth is growing upwards and the gum is not absorbed fast enough, injurious pressure is the consequence, which the surgeon's lancet removes. This unavailing "effort of Nature" is in more correct language an imperfect process of Nature, remediable by art. So with strangulated hernia, instead of saying that the surgeon "assists Nature to overcome the obstacle," it would surely be at once more correct and more simple to say that he removes it. "To act in accordance with the laws of Nature" comes then to this, that we should know the construction and action of the organism which we wish to rectify, that we should recognize the impediments, and remove them as delicately and tenderly as possible. If an engine is out of order, and the overseer goes about rashly twisting a screw here, drawing a bolt there, putting on the steam, taking it off again, in a hap-hazard fashion, we see at once that he is an ignorant bungler; but if he ascertains some particular part to be clogged or rusted, and clears away the obstacle, he acts, if you so please to term it, in accordance with the laws of the machinery. But there is no difference of *system* here. It is the difference between action founded upon knowledge, and action founded upon ignorance,—between rational therapeutics and blind empiricism.

The word *Nature* is the parent of many logomachies. Often it is a collective term, representative of all things around us, and of all their qualities and actions. It is also often used to express the causes of things being and doing as they are and do. When the mind fails to discover an efficient cause of an event, which is then an ultimate fact, we say it is the nature of the thing to be so—Nature has so ordered it; or, if we ascend to the First Cause of Nature, we say that it was the will of God that it should be so. But there is a strong instinct in the mind to interpose a power or intelligent agent in the production of events. Hence originated those hypotheses of φυσις, Archæus, anima regens, vital principle, &c., now vanished into the world of shadows. If this tendency is yielded to, philosophical investigation is continually liable to the risk of being cut short. The process of digestion may seem to be sufficiently explained by providing a *vis concoctrix*, and the complex congeries of actions in walking by a *vis ambulatrix*. If phrases of this kind are used only as provisional terms, pending the results of further inquiry, or for the mere purpose of classification, they may be very convenient; but, employed as expressions of causation, they cannot fail to do harm.

Dr. Combe, as we have seen, speaks of Nature as making efforts to do this, that, and the other; but I do not think he would say that, in teething, the epileptic paroxysms, the frightful laryngismus stridulus, and the slighter troubles of strangury and colic, are efforts of Nature. Yet they are not artificial: they are the morbid products of irritation in the cerebro-spinal axis, proceeding from the extremities of the trifacial nerve in the jaws. In strangulated hernia those terrible tormina, which add so much to the patient's sufferings, are the violent contractions of the tube, excited by its contents, which have been unduly retained in consequence of the mechanical impediment, and which become unusual stimulants to the muscular fibres, just as in the case of the bladder when some sudden stricture has occurred at the neck or in the urethra. Were there no obstacle, these "efforts," as Dr. Combe terms them, would be effectual in propagating the contents, but, as it is, they are prejudicial, and need to be quieted. But how came this obstacle? A fold of intestine slipped into the inguinal canal, the internal ring having been too patent, and some of the contents became gaseous and so distended the gut that it could not return by the narrow orifice through which it had entered. These events resulted from the natural constitution and properties of the parts, and we might therefore say that they were efforts of Nature. But, from long-established habits of thought, we recoil from such an expression. We instinctively transfer to external nature our own motives. A man has died of a wound; we find in the main artery, which had been divided, and through which his life had gushed away, an imperfect coagulum. We say that Nature had tried to save him, but had failed. We examine the trachea of a child who died of croup. The windpipe is all but entirely blocked up by the albuminous exudation, but we do not say, "Nature very nearly succeeded in choking the poor child." On the contrary, if we find never so small a portion of false membrane detached from the mucous lining, we exclaim, "See, Nature had made an effort to save the child." Or, take the case of a patient dead of typhoid fever. Death had been immediately induced by the hemorrhage from a Peyerian ulcer; on examining one of the elliptical patches in the ileum, we find that, at the base of the ulceration there is nothing but the thin layer of serous membrane. But we do not say, "Nature had very nearly killed the patient by spontaneous perforation." On the contrary, the least flake of albumen on the peritoneal covering of the gut would be enough to make us take it as a hint of Nature's kindly intention of strengthening the part by an adhesion to an adjoining surface. Such views occur to us, partly because the sanative purpose is ever uppermost in our minds, and partly because in normal anatomy and physiology we are familiar with most wonderful and extensive provisions for the safety and well-being of the living organism.

Nature is often put in opposition to disease. A morbid formation or disordered action is termed unnatural, which means that it is a deviation from the ordinary state or course of events in Nature. Yet, to show how *relative* the term is, we have only to point out that the very same action which, under some circumstances, would be pronounced unnatural, is, under other circumstances, termed natural. Suppuration in the brain around a tuberculous deposit is intensely morbid; suppuration of the subcutaneous cellular tissue in a boil or carbuncle is the natural mode of relief. Many analogous instances might be taken from hemorrhages; in fact, whenever in the course of disease any change takes place for the better, or tends to a favorable issue, if it is not obviously due to artificial interference, it is called a work or effort of Nature —Nature being so habitually associated in our minds with all that is good and beneficent. This association is so general as to indicate, were other proofs wanting, that there is a large preponderance of good in the existing state of things, and that evil and disease are exceptional. Still, *that they do exist* the philosopher is bound to concede. Imperfection of structure, incompleteness of action, tendency to decay, and downright destruction, meet us too obviously and too often to allow of our evading the conclusion that these faults, for some wise and in many respects unknown purpose, enter into the plan of the universe, just as the happy and tender affections in the lower animals are mixed up with the ferocities of beasts of prey and with the terrors of their helpless victims. One purpose, however, appears in this arrangement:—the opportunity offered for the play of man's intellectual resources. In the triumphs of his inventive faculties and laborious exertions over the defects and hard conditions of surrounding nature, he finds his most exalted gratification. The pleasure-grounds and the conservatories of the English gentleman show beauties of fruit and flower which uncultivated Nature can nowhere set forth; and, though the imagination may see the forms of Phidias imprisoned in blocks of marble, they would have remained there for ever had not the consummate artist set them free.

I am very glad that Dr. Combe is so anxious to do away with the impression that he would advocate a do-nothing system of treatment; but I repeat that I am quite at a loss to understand in what respect the treatment which he calls *natural* differs from the practice which has been taught and pursued by every one who founds his practice on a scientific acquaintance with the human frame in health and disease. I should certainly use a different phraseology. In the case of teething, all that is active on the part of Nature is tending to evil. Instead of aiding her exertions, you do your best to repress them, and you do that, the want of which in the work of Nature has been the cause of mischief. Of the treatment of fractures Dr. Combe has very just notions, and, instead of speaking so tenderly as he does on other occasions of "assisting Nature," I am glad that he ventures to talk of "rousing" her "to successful exertions." I agree with him also in thinking that you cannot put in a cement instead of the nutritive material out of which the new bone is to be formed, for this is one of the conditions necessary to the work in hand. No more can you fashion the marble statue without the marble,—or produce fine apples without the crab-tree. But the procuring of the marble, and even a chisel to boot, is but a small step in advance towards the shaping of a Pallas Athene, and there is a long and painful interval of labour and ingenuity between a sour crab and a Ribston pippin. The exudation-cells are essential to the union of the fractured bone, but they are not all. Let the man with his broken leg lie where he fell; his leg may be mended, but when he rises it is as likely as not to be two or three inches shorter than the other. Nay, let him be laid in bed, and Nature be aided, as Dr. Combe would say, by putting the limb in a good position; yet, unless it be confined by strong splints and bandages, it is very possible that some of "Nature's efforts" may consist of spasmodic twitchings, which jerk the ends of the bone out of place. Are these to be *aided* or *corrected?*

One remark is made by Dr. Combe, which surprises me greatly. I refer to what he says about removing a patient from the influence of a morbific cause, instead of allowing the cause to be still in operation, and only striving to counteract its effects. Can Dr. Combe really believe that any members of the profession are so inveterately stupid, or so unspeakably ignorant as to act in this fashion? Is there a practitioner to be found who, if he had the choice, would give bark to a patient in ague, instead of taking him away from the malarious district, though he might still require bark afterwards? or if the thorn were within reach of his forceps, would not make haste to pluck it out, instead of applying unguents and cataplasms? If Dr. Combe meets with such practitioners, I am sorry for him, "fallen upon evil days."—Alas! for the modern Athens! the city set on a hill! the city of the prophets. And can it be that with all the traditions and records of wisdom, from the days of Cullen and Gregory, and with all the light yet radiating from Alison, Christison, Simpson, and others, there are any medical minds within those walls, sunk in a state of such "total eclipse?" If so, it must be blindness from "excess of light."

Dr. Combe repudiates the idea of advocating a negative system of treatment in acute visceral inflammations. Though I was not replying to him in my former communication, I certainly had an impression that he had spoken very lightly about pleurisy, and very favorably of poultices, and milk and water; and on referring to his first letter, I find that impression to be not very far from the truth. Now I have seen many cases in which, because the patients had neglected to seek aid soon enough, or the disease had not been early recognized, Nature had enjoyed full and free play, and a dislocated heart and a lung solidified by compression were the consequences. Yet the patients had been lying in bed, had allayed their thirst with cooling drinks, and been comforted with all sorts of hygienic prettinesses.

It is unquestionable that some twenty years ago, even acute phlegmasiæ

were often treated with needless severity, and that blood was squandered most profligately by those who fastened their faith to the lancet only. But we have outlived those sanguinary days. Thanks to the help of those grand adjuvantia, calomel, opium, and antimony, it is long very long since we have seen a case requiring the repeated venesections common at the time of our noviciate. Peculiarities of constitution and other circumstances may compel us to pretermit the measure; but let the Paris and Vienna cases be what they may, I confidently appeal to the observant practitioners of this country, whether experience has not convinced them that if they are obliged to forego an early bleeding, the future management of the case becomes one of far greater difficulty than when it has been practised. I do not hesitate to say that the patients of a young practitioner may be endangered by the infusion of scepticism into his mind, as to the value of the orthodox treatment. For my own part I have an antipathy to the shedding of blood; it is the greatest possible relief to find that a case is one that does not require this remedy. Consider then what may be the effect of remarks, that insinuate a distrust of the necessity of active depletory measures on inexperienced practitioners with the same tendency, which from observation, I believe to be a very common one. In the early period of an illness, it is perhaps only from a nice balancing of indications that the inflammatory character of the disease is decided; but the patient shrinks from the lancet, the friends are averse to it, and now the doubts in the practitioner's mind, as to whether after all a bleeding is so very important, will turn the scale, and the bleeding is deferred. Twelve or fourteen, or, in country practice, twenty-four hours slip by before the next visit. And now the necessity is obvious enough, unless indeed the opportunity has been lost altogether. Imagine the case to be one not of pleurisy, but of peritonitis, and then judge what disastrous results ensue from the loss of time. I am sure I have my practical brethren with me when I express extreme jealousy of remarks, however enlightened may be their author, which tend to unnerve rather than to brace the young combatant in these life-and-death encounters.

I cordially acknowledge the great benefits conferred by Dr. Combe on the laity in those admirable works of his, on 'Physiology applied to Health,' on 'Digestion,' and on the 'Management of Infancy.' I doubt not that thousands gratefully associate with the name of Combe the formation of habits that have enabled them to be their own conservators of health, and to go through their daily duties with ease and cheerfulness. But when Dr. Combe comes forward to lecture the profession on the importance of *studying Nature*, I beg leave to tell him that though Lord Bacon has been dead more than two centuries, he yet speaks, and in tones more intelligible and commanding than ever; that his sublime testamentary prediction has been long fulfilled; that not only did the "next ages" reverently take up his name and words, but that every succeeding age has resounded them yet louder and louder; that they are lisped by children, and syllabled by strange tongues on

"Sands, and shores, and desert wildernesses,"

and that no class of practical philosophers have more obediently remembered the words of the great master, than the followers of medicine from the days of Sydenham and Baglivi downwards. Dr. Combe has this time mistaken his mission; this is not his rôle; and we must bid him *"sui plausu gaudere theatri."*

I remain, my dear Sir, yours very sincerely,

J. A. Symonds.

